# Seed Layer Optimisation for Ultra-Thin Sb_2_Se_3_ Solar Cells on TiO_2_ by Vapour Transport Deposition

**DOI:** 10.3390/ma15238356

**Published:** 2022-11-24

**Authors:** Remigijus Juškėnas, Arnas Naujokaitis, Audrius Drabavičius, Vidas Pakštas, Deividas Vainauskas, Rokas Kondrotas

**Affiliations:** Center for Physical Sciences and Technology, Sauletekio Ave. 3, 10257 Vilnius, Lithuania

**Keywords:** Sb_2_Se_3_, thin film solar cells, VTD, seed layer

## Abstract

Antimony selenide (Sb_2_Se_3_) material has drawn considerable attention as an Earth-abundant and non-toxic photovoltaic absorber. The power conversion efficiency of Sb_2_Se_3_-based solar cells increased from less than 2% to over 10% in a decade. Different deposition methods were implemented to synthesize Sb_2_Se_3_ thin films, and various device structures were tested. In search of a more environmentally friendly device composition, the common CdS buffer layer is being replaced with oxides. It was identified that on oxide substrates such as TiO_2_ using vacuum-based close-space deposition methods, an intermediate deposition step was required to produce high-quality thin films. However, little or no investigation was carried out using another very successful vacuum deposition approach in Sb_2_Se_3_ technology called vapour transport deposition (VTD). In this work, we present optimized VTD process conditions to achieve compact, pinhole-free, ultra-thin (<400 nm) Sb_2_Se_3_ absorber layers. Three process steps were designed to first deposit the seed layer, then anneal it and, at the final stage, deposit a complete Sb_2_Se_3_ absorber. Fabricated solar cells using absorbers as thin as 400 nm generated a short-circuit current density over 30 mA/cm^2^, which demonstrates both the very high absorption capabilities of Sb_2_Se_3_ material and the prospects for ultra-thin solar cell application.

## 1. Introduction

Photovoltaic technology is considered one of the best strategies to achieve environmentally benign energy production. Thin-film solar cells have advantages over common silicon-based solar cells such as being lightweight and having low production costs. The most advanced thin-film solar cell technologies, such as CIGSe (copper indium gallium diselenide), CdTe (cadmium telluride) and perovskites have reached PCE (power conversion efficiency) records higher than 22% [1,2]. However, there are major drawbacks to the mentioned technologies: the price and scarcity of indium and the toxicity of cadmium do not make them environmentally or cost-friendly, and perovskite solar cells still have an unresolved issue with thermodynamic stability under ambient conditions [3]. Antimony selenide (Sb_2_Se_3_) has emerged as an alternative material for solar cell absorbers because of its high absorption coefficient (>10^5^ cm^−1^), a suitable band gap of 1.1–1.3 eV and *p*-type conductivity under Se-rich conditions [4]. In addition, Sb_2_Se_3_ is a non-toxic, environmentally benign, highly stable compound made up of Earth-abundant elements [4].

Over the past decade, the PCE of Sb_2_Se_3_ solar cells has increased from <2% to 10.1% [5]. Various synthesis methods and buffer layers have been tried out to optimize the structure of Sb_2_Se_3_ solar cells and improve their performance [6,7,8]. While the highest PCE was achieved using CdS, the toxicity issue of Cd incentivized a search for alternative buffer layers. Since oxide materials such as ZnO, SnO_2_, and TiO_2_ are free from toxic elements, they proved to be suitable partner layers and have been successfully implemented in Sb_2_Se_3_ solar cells [8,9,10,11]. Among all oxide buffer layers, the highest PCE has been demonstrated using TiO_2_ [12]. However, because of the inert nature and strong Ti–O bond in TiO_2_, the deposition of the Sb_2_Se_3_ films on TiO_2_ is more complicated. For example, using the CSS (close space sublimation) or RTE (rapid thermal evaporation) methods to deposit Sb_2_Se_3_ on TiO_2_ required a multiple-stage deposition process [13] (unlike on the CdS) [14]. The challenge is that the nucleation energy barrier on TiO_2_ is larger than on other substrates, which can lead to a high density of pinholes and/or a non-planar film morphology. It was recognized that a seed layer is necessary to produce high-quality Sb_2_Se_3_ thin films on TiO_2_ substrates. Using the CSS approach, Hutter et al. introduced a low-source-temperature (T_src_ = 350 °C) step to deposit the seed layer [15]. This allowed the achievement of the pinhole-free absorber, and >6% of PCE devices were demonstrated. Using the CSS approach, Krautmann et al. expanded the study on the seed layer’s effect on grain orientation, defects, and solar cell performance [16]. They showed that the seed layer was crucial for promoting favourable grain orientation and increasing PCE. Using an RTE, Li et al. introduced an additional seeds-screening step to induce the growth of grains with only (hkl); where l ≠ 0 orientation and in optimized device structure, a 7.62% PCE was achieved [13]. However, only very few (if any) reports about the formation of seed layer or Sb_2_Se_3_ absorber on TiO_2_ using vapour transport deposition (VTD) have been published. VTD is an industrially scalable deposition method which has been proven to be very successful in the fabrication of high-efficiency Sb_2_Se_3_ solar cells on the CdS buffer layer [17]. However, a multiple-stage deposition process is more difficult to realize in a VTD setup because of equipment limitations where slower temperature ramps are used and the substrate temperature is controlled indirectly [18].

In this work, we present an optimized three-step deposition protocol to obtain high-quality, compact, and pinholes-free Sb_2_Se_3_ thin films on TiO_2_ substrates using the VTD method. Various experimental parameters were changed to obtain the desired properties of the seed layer and the final Sb_2_Se_3_ absorber. Since a high temperature difference between the source and substrate could be fulfilled in the VTD setup, compact, pinholes-free seed layers were achieved already at a thickness of <100 nm. This allowed producing absorbers as thin as 400 nm and fabricating ultra-thin solar cells which generated an over 30 mA/cm^2^ short-circuit current density. The effect of absorber thickness on Sb_2_Se_3_ solar cell performance was studied, and the champion cell achieved 4.8% PCE. The results of this work provide a technological pathway for seed layer formation and reveal the VTD method’s high potential for fabricating ultra-thin Sb_2_Se_3_ solar cells.

## 2. Materials and Methods

TEC15-type FTO was used as a substrate (GreatCell Solar Materials, Queanbeyan, Australia), which, before buffer layer deposition, was cleaned with detergent followed by ultrasonication in ethanol and deionized water for 5 min each. A compact 20–30 nm layer of titanium dioxide (TiO_2_) with an anatase structure was deposited at 465 °C via spray pyrolysis from a solution composed of acetylacetone (200 µL) and titanium isopropoxide (300 µL) dispersed in ethanol (4.5 mL).

Sb_2_Se_3_ thin films were deposited using a three-zone tube furnace to realize a vapour transport deposition (VTD) approach (Supplementary Materials, Figure S1). Sb_2_Se_3_ powder (99.99% Shanghai Xinglu Chemical Co., Shanghai, China) was used as source material. About 2 g of powder was weighed which was enough to keep approximately 3.2 cm^2^ area of powder in the ceramic crucible and run at least five consecutive depositions. Source powder was placed in the centre of heating zone 2, whereas the substrate was located at varying distances outside of heating zone 1 (Supplementary Materials, Figure S1). The pressure in the tube was achieved using rotary pump and during deposition process was in 0.6–1.2 Pa range. The top temperature of heating zones 1 and 3 was fixed at 420 °C with a ramp rate of 20 °C/min, and only zone 2’s heating parameters were varied (Figure S2). Samples were positioned on the graphite support in an almost vertical position (80 degrees) with respect to the tube axis (Supplementary Materials, Figure S1). The range of tested heating parameters of zone 2 as well as the optimum are shown in Figure S2 of Supplementary Materials. In brief, it comprised three stages: (i) deposition of the seed layer; (ii) annealing of the seed layer; and (iii) deposition of the absorber.

Solar cells were formed by thermally evaporating gold on top of the Sb_2_Se_3_ absorber through a mask in the vacuum chamber of VAKSIS PVD Vapor-5S_Th (Vaksis R&D and Engineering, Ankara, Turkey). The final solar cell structure was superstrate (FTO/TiO_2_/Sb_2_Se_3_/Au) with a cell size of 12.5 mm^2^ defined by gold contact area. Gold electrodes were covered with either silver or carbon paste to protect cells from puncturing when contacting for *I–V* measurements.

The surface morphology and elemental composition of thin films were carried out in a dual-beam system of a scanning electron microscope FEI Helios Nanolab 650 (Eindhoven, The Netherlands) equipped with an EDX spectrometer from Oxford Instruments. Cross-sections were made with a 30 keV Ga ions beam.

Film structure measurements were performed using a SmartLab diffractometer (Rigaku, Tokyo, Japan) equipped with a 9 kW rotating Cu anode X-ray tube. X-ray diffractograms were recorded in the Bragg–Brentano scanning geometry with a 0.02° step size. Crystalline phases were identified by using the diffraction database PDF4+. The diffractometer was tested and calibrated using a certified standard LaB_6_ SRM 660b powder.

The current-voltage (*I-V*) characteristics of Sb_2_Se_3_ solar cells were measured under standard test conditions (AM 1.5G, 100 mW/cm^−2^) using a Newport Oriel Class AAA solar simulator. Keithley 2400 was used as a source meter to record *I-V* characteristics.

Capacitance-voltage (*C-V*) characteristics were calculated from voltage-dependent impedance measurements using potentiostat-galvanostat Autolab PGSTAT302 (Metrohm AG, Herisau, Switzerland) in 1–100 kHz frequency range and at 10 mV voltage steps. Capacitance was calculated from the imaginary part of the measured impedance.

Sb_2_Se_3_ thin-film absorbance was measured using double-beam JASCO V-670 UV-VIS spectrophotometer (Pfungstadt, Germany) in 300 – 1500 nm range at room temperature.

## 3. Results

### 3.1. Optimization of the Seed Layer

**Figure 1:**
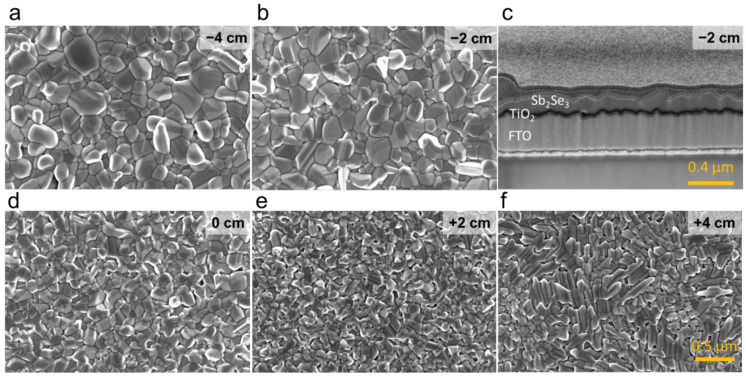
Top-view (**a**,**b**,**d**–**f**) SEM micrograph of Sb_2_Se_3_ seed layer deposited at different sample heating zone distances indicated in the image. (**c**) SEM image of FTO/TiO_2_/Sb_2_Se_3_-seed layer structure cross-section deposited at *d* = −2 cm.

**Figure 2:**
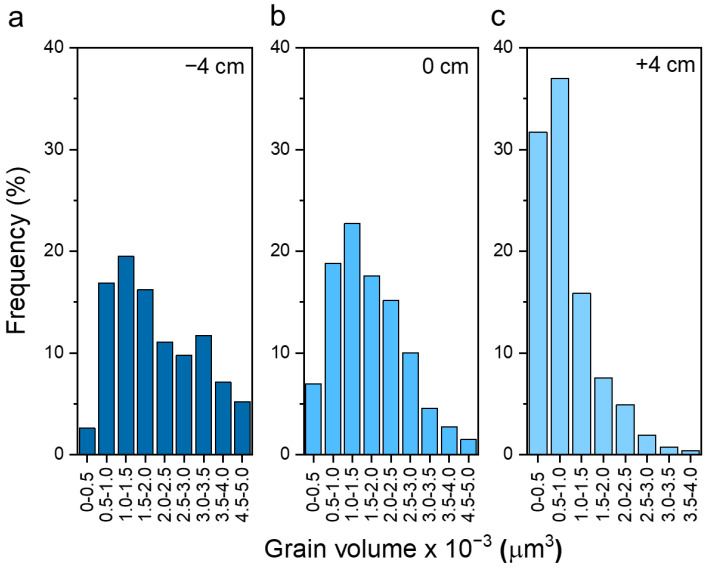
Grain size distribution in Sb_2_Se_3_ seed layers deposited at (**a**) −4 cm, (**b**) 0 cm, and (**c**) +4 cm distances between zone edge and sample. Grain volume was calculated by multiplying seed layer thickness with measured grain surface area estimated from SEM image.

**Figure 3:**
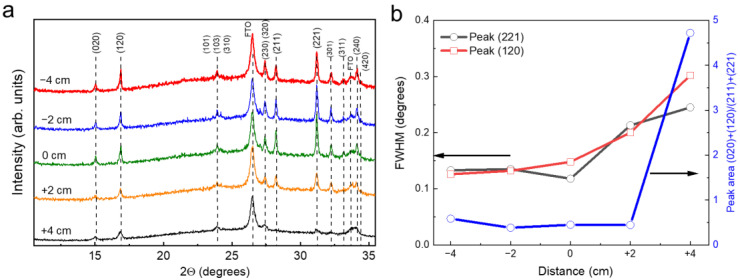
(**a**) XRD patterns of Sb_2_Se_3_ seed layer deposited at different sample heating zone distances. All XRD peaks were assigned to orthorhombic Sb_2_Se_3_ phase (PDF 01-089-0821) and SnO_2_ (PDF 00-041-1445). (**b**) FWHM and ratio of (020) + (120) peak area to (221) + (211) as a function of sample heating zone distance.

As was found by other authors, the seed layer is a critical step in obtaining high-quality Sb_2_Se_3_ thin films and increasing deposition reproducibility on the TiO_2_ buffer layer [19]. Therefore, we first examine how VTD deposition parameters affect the properties of the seed layer. We varied source temperatures (*T_src_*), ramp rates, substrate heating zone distance (*d*), and annealing duration to find optimal conditions for seed layer deposition. Key criteria for the seed layer are: (i) full coverage of the substrate surface; (ii) high crystallinity; and (iii) favourable grain orientation (i.e., hkl, where l ≠ 0). To obtain high surface coverage, it is important to keep a large temperature difference between source and substrate temperatures because it promotes high-density nucleation. For this reason, we used a large graphite support, which acted as a heat sink and enabled a slow increase in *T_sub_*. On contrary, *T_src_* followed the heating zone temperature profile where the fastest ramp was applied because of the low heat capacity and the mass of the crucible (Supplementary Materials, Figure S2). In this way, a large temperature difference is achieved between *T_src_* and *T_sub_*. It is important to note that the sample’s distance from the heating zone will also influence *T_sub_* significantly and is often used to control the substrate temperature in the VTD setup [14]. We also found that among the tested deposition parameters, *d* was one of the most important ones. The change in *T_src_* in the first deposition stage mainly affected the thickness of the seed layer and, to some extent, grain orientation (Figure S3). However, when using low *T_src_*, the deposition of the seed layer was much more sensitive to pressure fluctuations; therefore, we chose a higher end of *T_src_* for further study. Keeping all other deposition parameters fixed, we focused our study on structural seed layer properties with respect to *d* only. We varied *d* from −6 to +4 cm, where 0 cm indicates that the sample was placed right at the edge of the heating zone, and negative (positive) values denote how much samples were introduced inside (outside) the heating zone (higher/lower *T_sub_*) (Figure S1). The variation in the seed layer morphology as a function of *d* is presented in Figure 1. Note that at *d* = −6 cm, the seed layer started to re-evaporate because *T_sub_* was too high, and no seed layer was present at the end of the annealing step. We found that, in all cases, we obtained compact and pinhole-free seed layers. When *d* < 0 cm, the layers were composed of rather large round grains and size distribution was dispersed (Figure 2a). This is indicative of the ripening effect when smaller grains are consumed to grow larger ones, therefore leading to a broader size distribution [20]. As *d* was increased to positive values, grains became smaller and had narrower size distributions (Figure 2b,c). This can be expected because when *T_sub_* is reduced, the adatom diffusion length on the surface decreases, which leads to the formation of smaller grains [16]. Note that at *d* = +4 cm, the shape of the grains changed completely from round to elongated. As will be shown later, this coincided with a substantial change in grain orientation (Figure 3b). Note that the thickness of the seed layer depended on *d* only when *d* > 0 cm (Figure S4), which signified that the growth rate gradually decreased as the sample was moved outside of the heating zone. This occurred because more Sb_2_Se_3_ vapour condensed on the quartz walls and less reached the substrate when it was moved away from the heating zone. Changing the distance to positive values, therefore, had a two-fold effect: a decrease in substrate temperature and a reduced deposition rate.

Sb_2_Se_3_ seed layer phase composition and grain crystallographic orientation was studied using XRD. All peaks were assigned to the orthorhombic phase of Sb_2_Se_3_ (PDF #01-089-0821) and FTO (PDF #00-041-1445) whose reflections originated from the substrate. Phase composition did not depend on the sample heating zone distance (Figure 3a). However, the full width at half maximum (FWHM) did correlate with the distance: FWHM decreased exponentially as the distance changed from positive to negative (Figure 3b). This is a consequence of gradually increasing substrate temperature when the sample is positioned inside the heating zone. This also means that the crystalline quality of the seed layer improved as *T_sub_* was increased, although it started to saturate at *d* < 0 cm. Note that while grain size estimated from SEM was increasing when *d* was changed to −4 cm, the FWHM of XRD peaks did not change substantially from 0 to −4 cm. This shows that grain size is not an indication of crystalline quality. XRD peak FWHM is related to the size of the crystalline domain, which is often wrongly directly compared with the grain size.

Grain orientation was estimated by taking the ratio of the peak area of the most intensive XRD peaks of planes that are orientated with a ribbon axis parallel to the surface, i.e., (020) and (120), and planes where the ribbon axis is at a certain angle, such as (211) and (221). Note that the Voigt function was used for fitting XRD peaks and calculating the peak area and FWHM. We found that grain orientation did not depend heavily on the distance in the −4–+2 cm range, but a stark change occurred at +4 cm. It also coincided with a change in grain shape (Figure 1f); therefore, the elongated nanorod-like shape of the grains was indicative of a ribbon parallel to the surface orientation and was also observed by other authors [14,16,21]. This shows that under low deposition rates and substrate temperatures, grains tend to orient themselves in a way that maximizes the van der Waals surface area.

Based on the criteria for the seed layer, we found that the optimal *d* was in the −4–0 cm range. In this region, deposited seed layers were compact, fully covered the substrate surface, were of high crystalline quality (in terms of FWHM) and had favourable grain orientations. For the Sb_2_Se_3_ absorber deposition, we selected −2 cm as the optimal distance. The reason is that, at −4 cm, there is a higher risk of seed layer re-evaporation, whereas at 0 cm, it required a longer annealing step duration to increase *T_sub_* for the absorber deposition.

### 3.2. Solar Cell Fabrication and Performance

**Figure 4:**
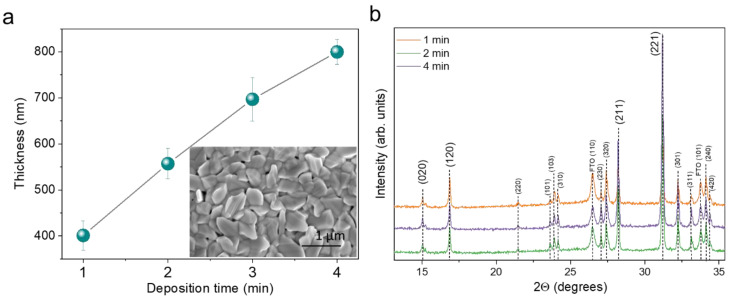
(**a**) An average absorber thickness determined from cross-sectional SEM images as a function of deposition time in the last process step. Inset—top-view SEM image of Sb_2_Se_3_ absorber deposited at 3 min. (**b**) XRD patterns of Sb_2_Se_3_ absorbers grown at different deposition times. All XRD peaks were assigned to orthorhombic Sb_2_Se_3_ phase (PDF 01-089-0821) and SnO_2_ (PDF 00-041-1445).

Having identified optimal seed layer deposition conditions, the complete Sb_2_Se_3_ absorber was grown on the top of the seed layer in the last deposition process step (Figure S2a). This included ramping the *T_src_* up to 520–525 °C and maintaining it for a fixed period. The top annealing temperature duration varied from 1 to 4 min, which resulted in the final absorber’s thickness being in the 400–800 nm range. Note that thickness variation did not follow deposition time linearly (Figure 4a), and as can be seen, the growth rate was higher in the first few minutes and then started to decline. We believe this was related to the initial *T_src_* overshoot (Figure S2), which always occurs with the resistive-type furnaces when a high-temperature ramp rate is used. The surface morphology of the Sb_2_Se_3_ layer did not depend on the deposition time (Figure S5) and remained almost the same, as shown in the inset of Figure 4a. The phase composition of the absorber was the same as that of the seed layer—orthorhombic Sb_2_Se_3_. The grain orientation did not change significantly with absorber thickness, although (hkl, l ≠ 0) planes were more prominent than in the seed layer (Figure 4b). Chemical composition was slightly deposition-time-dependent. The longer the deposition time, the more an Sb-rich composition was observed (Table S2). However, the trend was very small (<1 at. %); therefore, we do not expect it to have a significant influence on solar cell performance. Overall, the structural properties of the Sb_2_Se_3_ absorber were almost identical, except for thickness. This then allowed us to study how the thickness of the absorber rather than structural characteristics affected the solar cell performance.

**Figure 5:**
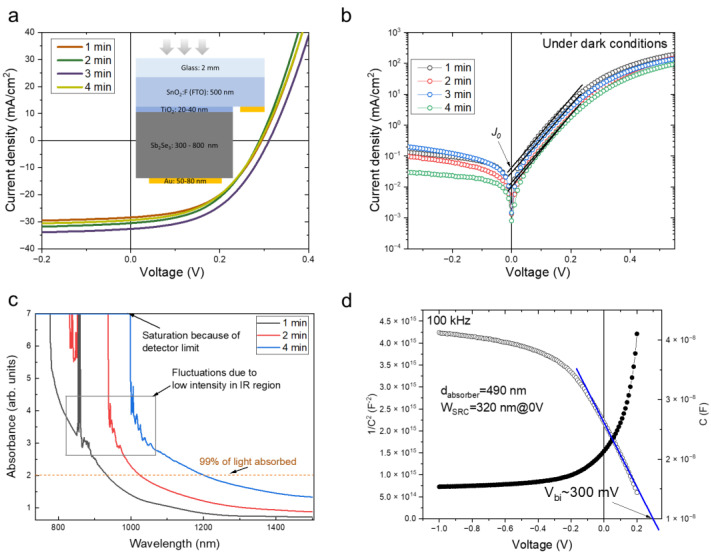
Light (**a**) and dark (**b**) *J*-*V* curves of champion Sb_2_Se_3_ solar cells fabricated at various deposition times. (**c**) Absorbance spectra of Sb_2_Se_3_ solar cells synthesized at different deposition times. (**d**) *C*-*V* and 1/C^2^ curves measured at 100 kHz frequency under dark conditions of the Sb_2_Se_3_ solar cell with an absorber thickness of 490 nm.

The final solar cell structure was FTO/TiO_2_/Sb_2_Se_3_/Au (Figure 5a, inset). *J-V* curves of the champion cells for each case are depicted in Figure 5a, and average performance parameters are listed in Table 1. We see that power conversion efficiency (PCE) varied in the 3.4–4.6 % range and was rather dispersed within a sample. The largest variation was observed in short-circuit current density, *J_SC_* (Table 1). We speculate that it was related to the uneven surface oxidation, which is typical effect when using low-vacuum conditions such as in the VTD process [22]. In another batch of fabricated solar cells under very similar conditions, we applied surface etching using a dilute (NH_4_)_2_S solution. This not only reduced scattering in solar cell parameters but also increased the fill factor (FF) (Table S1). On the other hand, the peak-performing cells all showed rather similar solar cell parameters with a record efficiency of 4.9% (Table 1). There are very few studies where Sb_2_Se_3_ solar cells are deposited on Cd-free buffer layers using the VTD approach indicating a non-trivial deposition protocol on oxide buffer layers. Using the same deposition method (VTD) on double-oxide buffer layers (SnO_2_/TiO_2_), Wang et al. demonstrated 4.7% PCE, which was boosted to 5.8% using SbCl_3_ surface treatment [23]. A surprising observation was that the generated *J_SC_* did not depend on the absorber thickness, even when it was increased two times. Neglecting limited charge carrier diffusion length, usually, the thicker the absorber, the more photons are absorbed and converted to electron–hole pairs, leading to increased *J_SC_*. For example, to absorb all solar radiation above the bandgap, 1.5–2 µm-thickness absorbers are required for well-established thin-film solar cell technologies such as Cu(In, Ga)Se_2_ [24] and Cu_2_SnZnSe_4_ [25], and even higher thicknesses are needed for CdTe [26]. In the case of the Sb_2_Se_3_ absorber, a *J_SC_* over 30 mA/cm^2^ was generated using layers as thin as 0.4 µm and without any intentional light-management solutions. The measured absorbance of Sb_2_Se_3_ films showed that even in the thinnest sample, 99% of incident radiation was absorbed at a 950 nm wavelength and below (Figure 5c). This demonstrates the high absorption capabilities of Sb_2_Se_3_ and its potential application in ultra-thin solar cells. Using the experimentally measured absorption coefficient of Sb_2_Se_3_, we calculated the theoretical limit of *J_SC_* at a specific absorber thickness using an idealized p–n junction (Table 1). The sample with an absorber thickness of 0.4 µm generated 86% of the theoretical *J_SC_*, and this is not considering reflection losses from a glass substrate. However, with an increasing absorber thickness, *J_SC_* did not improve, which suggests that collection efficiency decreased accordingly. Carrier mobility and carrier lifetime are rather low in Sb_2_Se_3_ [27], leading to a short carrier diffusion length. Once absorber thickness exceeds carrier diffusion length, carriers recombine before reaching contacts and current is lost as heat. Another limiting factor in our solar cells was a low open-circuit voltage (*V_OC_*), which was in the 290–300 mV range. For state-of-the-art Sb_2_Se_3_ solar cells, the *V_OC_* is >400 mV. In our case, a Fermi-level pinning is suspected at the Sb_2_Se_3_–TiO_2_ interface because the built-in voltage (*V_bi_*) determined from *C-V* measurements (Figure 5d) was the same as the *V_OC_*. The *V_bi_* is equal to the Fermi-level difference of *n*- and *p*-type semiconductors before forming a heterojunction, and from the data available in the literature, the *V_bi_* is expected to be somewhere from 450 to 650 mV, depending on the doping level in Sb_2_Se_3_ and the approach used for the synthesis of TiO_2_ [28,29]. Therefore, we suggest that the low *V_bi_* (which also limited *V_OC_*) was because of Fermi-level pinning likely caused by interface defects. The measured *J-V* curves under dark conditions also indicated that severe recombination was present in the device. For a state-of-the-art Sb_2_Se_3_ device, dark currents under negative bias usually fall below 10^−2^ mA/cm^2^, whereas the dark saturation current density (*J_0_*) is below 10^−3^ mA/cm^2^ [30,31]. In the samples under study, *J_0_* was in the 1 × 10^−2^–4 × 10^−2^ mA/cm^2^ interval (Figure 5b). Such a high dark saturation current is an indication of a high recombination rate that can originate from the interface and bulk defects. We believe that interface defects were the primary source for high *J_0_* because of the pinned *V_OC_*.

## 4. Conclusions

In this work, we use the vapour transport deposition method (VTD) to fabricate Sb_2_Se_3_ solar cells. One of the main advantages of this method is that the growth rate can be controlled across a wide range, which enables us to obtain very thin, but compact, conformal and pinhole-free films. Additionally, because of the lower growth rate and great distance between the source and substrate, the high substrate temperature is not required in contrast to close-space sublimation or rapid thermal annealing approaches. This makes VTD compatible with flexible organic substrates. Herein we demonstrated that solar cells can be fabricated using an optimized three-step deposition protocol as thin as 0.4 µm Sb_2_Se_3_ and showed that short-circuit current density over 30 mA/cm^2^ was reached. This shows a high-absorption coefficient and the potential of Sb_2_Se_3_ for ultra-thin solar cell applications.

## Figures and Tables

**Table 1:** Device performance parameters and absorber thickness of Sb_2_Se_3_ solar cells fabricated at different deposition durations. Provided parameters are averages with an error estimated as standard deviation.

Deposition Time	*J_SC_*, mA/cm^2^	*V_OC_*, mV	FF, %	PCE, %	Thickness, nm	Limit *J_SC_*, mA/cm^2^
1 min Peak cell	29.4 ± 1.6 31.6	286 ± 6 282	47.8 ± 0.8 48.6	4.0 ± 0.25 4.33	400 ± 32	36.5
2 min Peak cell	27.2 ± 3.9 30.5	282 ± 8 289	47 ± 1.0 47.9	3.6 ± 0.6 4.23	557 ± 33	38.1
3 min Peak cell	30.2 ± 1.8 32.6	316 ± 7 311	48.4 ± 0.5 47.8	4.6 ± 0.2 4.86	697 ± 47	39.1
4 min Peak cell	24.5 ± 3.4 29.4	292 ± 2 292	47.6 ± 0.5 47.2	3.4 ± 0.45 4.05	800 ± 27	39.7

## Data Availability

Data are contained within the article or supplementary material. The data presented in this study are available in the supplementary file.

## Supplementary Materials

The following supporting information can be downloaded at: https://www.mdpi.com/article/10.3390/ma15238356/s1. Supplementary files include one document with Figure S1 (schematic representation of VTD setup); Figure S2 (heating profiles during deposition process); Figure S3 (SEM and XRD of seed layers); Figure S4 (SEM cross-sectional images of seed layers); Figure S5 (SEM top-view and cross-sectional images of Sb_2_Se_3_ solar cells); Table S1 (solar cell parameters); Table S2 (chemical composition of Sb_2_Se_3_ absorber) and raw files of XRD, I-Vs and SEM.
