# Supplementary material for: Seed Layer Optimisation for Ultra-Thin Sb2Se3 Solar Cells on TiO2 by Vapour Transport Deposition
Source: Materials (Basel). 2022 Nov 24;15(23):8356. doi: 10.3390/ma15238356 (PMC9739395; doi:10.3390/ma15238356)
Supplement: Supplementary file 1 [file materials-15-08356-s001.zip › materials-2013864-supplementary.pdf]

Seed layer optimisation for ultra-thin Sb_2Se_3 solar cells on TiO_2 by vapor transport deposition

*Remigijus Juškėnas, Arnas Naujokaitis, Audrius Drabavičius, Vidas Pakštas, Deividas Vainauskas, Rokas Kondrotas**

Center for Physical Sciences and Technology, Sauletekio ave. 3, Vilnius, 10257, Lithuania

**corresponding author; rokas.kondrotas@ftmc.lt*

Vapour transport deposition setup was realised in three-zone tube furnace. Source material is placed in the heating zone 2 (Z2) whereas Z1 and Z3 act as vapour transporting zones. Sample is placed at certain distance from heating zone Z3 downstream. The temperature of Z1 and Z3 was selected as such that no condensation of Sb_2Se_3 vapour takes place in these zones. Also, the temperature of Z3 affects substrate temperature. In our case the optimal temperature for Z1/Z3 was found to be 420 °C (Figure S2).

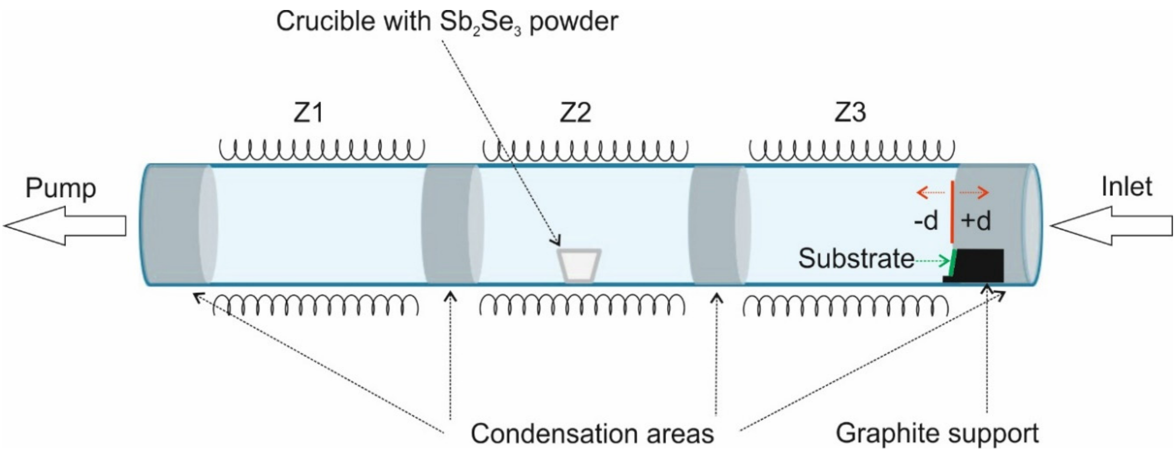

Figure S1. Schematic representation of VTD deposition setup.

In Figure S2a a schematic representation of temperature profiles of three-stage deposition process is shown and the range of parameters tested. Schematic presentation of optimal conditions that were used to synthesize Sb_2Se_3 thin film are shown in Figure S2b. Recorded temperature readings of the furnace thermocouples are shown in FigureS2c. An overshoot can be observed at top T_{src} temperatures because of resistive type heating elements. Thermocouple readings of the graphite support which reflect substrate temperature are shown in Figure S2b as black and grey curves. It was recorded under ambient and low vacuum (10^2 Pa) conditions. Lower substrate temperature under vacuum is a result of reduced heat transfer by the convection.

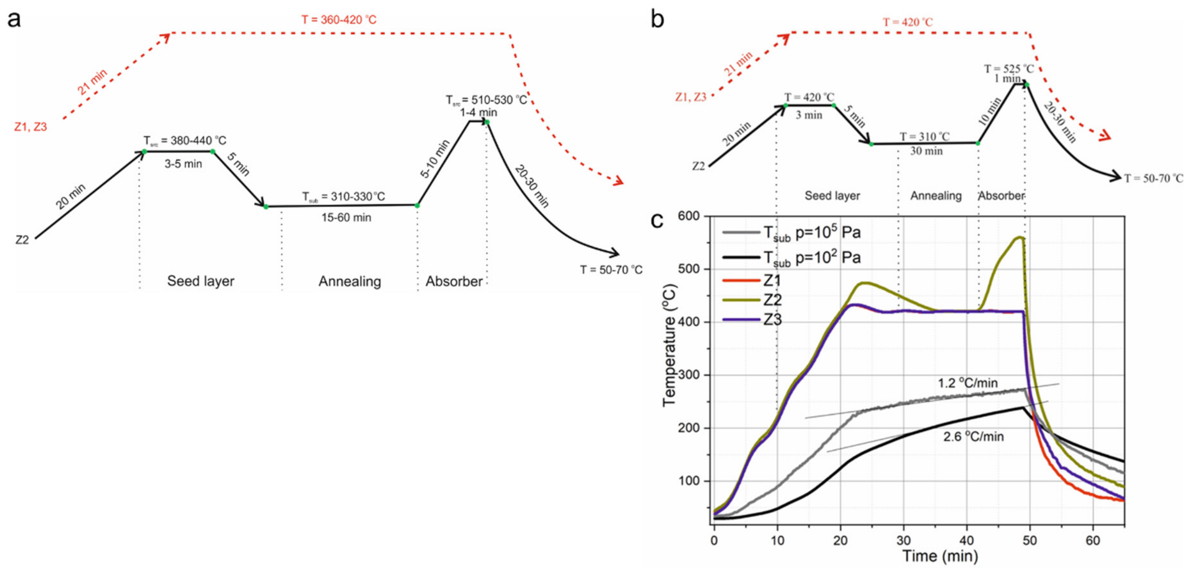

Figure S2. Schematic representation of temperature profiles of (a) tested conditions and (b) optimal deposition conditions. c) Thermocouple temperature readings of each zone and graphite support.

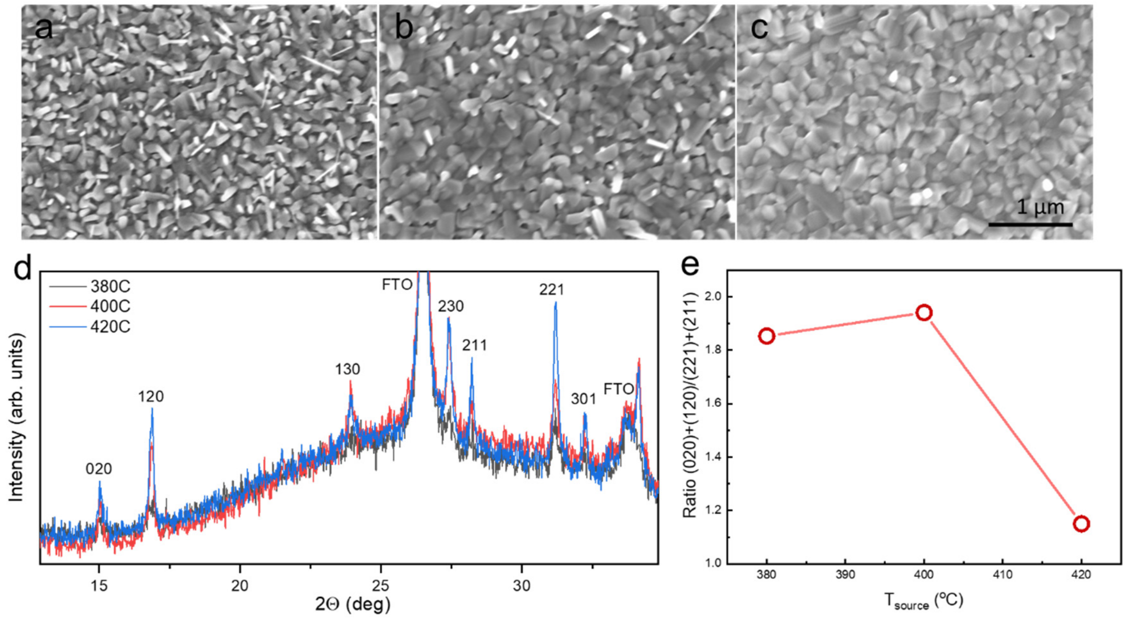

Figure S3. Top-view SEM images of Sb_2Se_3 seed layer deposited at different source temperature (a) 380 °C, (b) 400 °C, (c) 420 °C. (d) XRD patterns of seed layer deposited at various source

temperature. (e) calculated XRD peak area ratio of planes (120), (020) to (221), (211) as a function of source temperature.

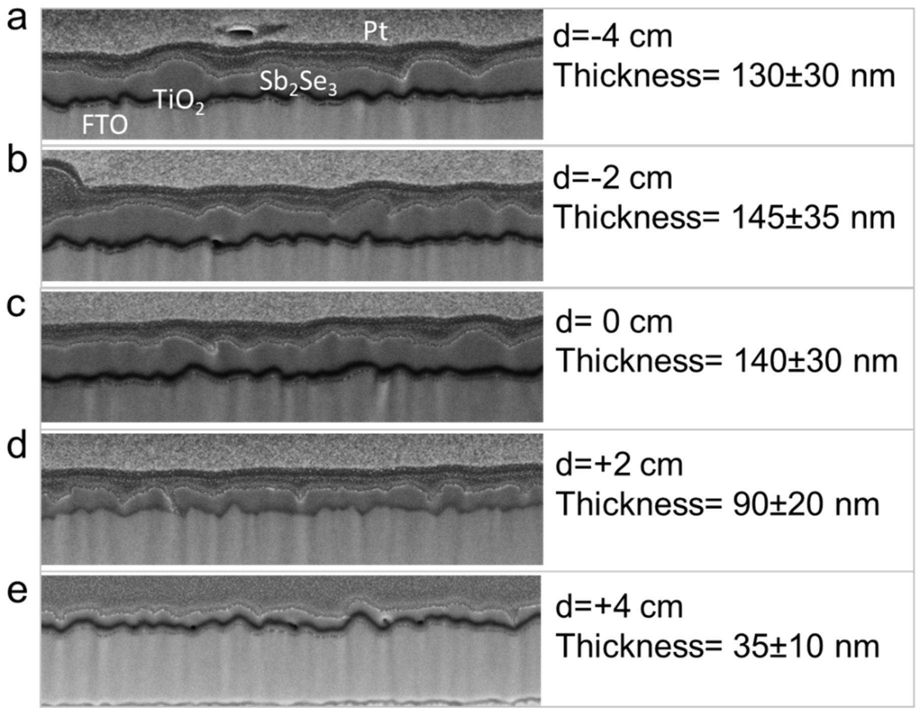

Figure S4. Cross-sectional SEM images of Sb_2Se_3 seed layers deposited at different heating zone-substrate distances, d. Pt is deposited during preparation of cross-section by ion beam milling.

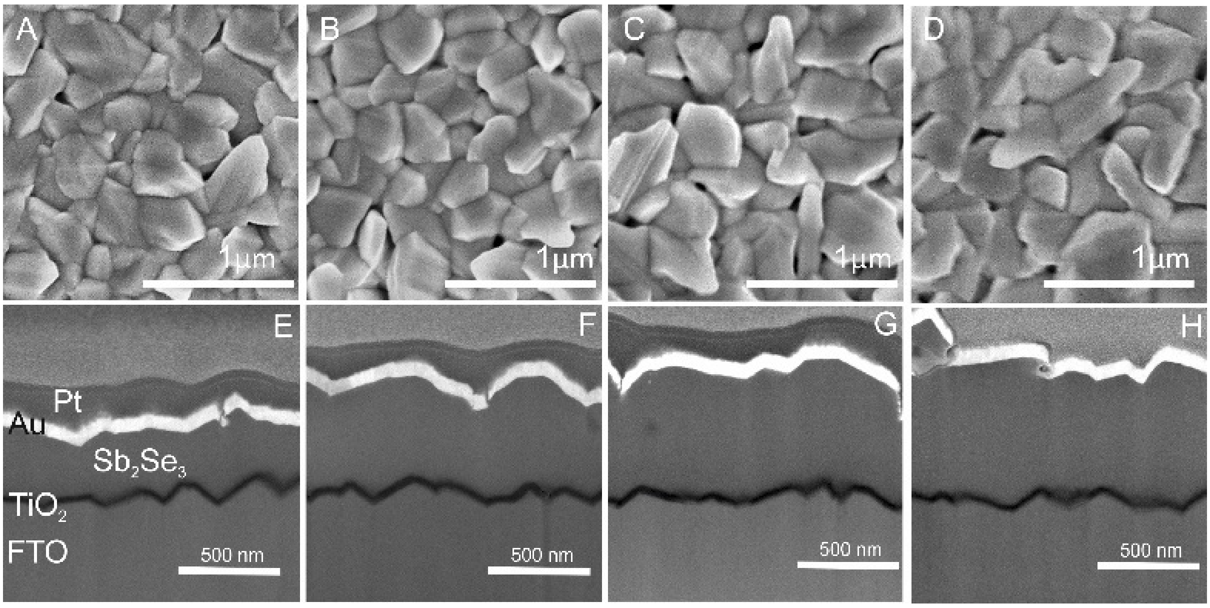

Figure S5. (A-D) top view and (E-H) cross-sectional SEM images of Sb_2Se_3 solar cells deposited at different duration (A,E) – 1 min, (B, F) – 2 min, (C, G) – 3 min; (D, H) – 4 min. Pt was deposited for focused ion beam milling purposes.

A series of samples presented in Table S1 have slightly modified deposition recipe for seed layer in contrast with those mentioned in the main paper. However, under optimal cleaning conditions (c=0.03 mmol/l for 120 s), solar cell parameters were significantly improved and more uniform.

Table S1. Sb_2Se_3 solar cell parameters with applied surface etching in $(NH_4)_2S$ solution

**Sample**	**$(NH_4)_2S$ concentration**	**Time, s**	**PCE, %**	**V_{oc}, mV**	**J_{sc}, mA/cm^2**	**FF,%**
R46, Ref.	As-deposited	0	3.41±0.08	304±5	21.5±0.15	52±0.7
R51	0.3 mmol/l	5	4.58±0.22	303±3	29.2±1.6	51±0.2
R53	0.3 mmol/l	30	4.08±0.47	296±4	27.7±1.3	49±3
R45	0.3 mmol/l	60	3.42±0.63	289±9	25.6±1.7	45.7±4.2
R44	0.03 mmol/l	60	4.08±0.35	297±3	26.9±2.3	51.2±0.5
R50	0.03 mmol/l	120	4.96±0.07	317±1.6	29.6±0.15	52.8±0.6

Table S2. Chemical composition of Sb_2Se_3 absorbers deposited at different deposition durations.

**Sample**	**Sb, at%**	**Se, at%**	**[Se]/[Sb]**	**3[Sb]/2[Se]**
1 min	41.7	58.3	1.40	1.07
2 min	42.0	58.0	1.38	1.09
3 min	42.3	57.7	1.36	1.1
4 min	42.5	57.5	1.35	1.11
